# Vypal2: A Versatile Peptide Ligase for Precision Tailoring of Proteins

**DOI:** 10.3390/ijms23010458

**Published:** 2021-12-31

**Authors:** Dingpeng Zhang, Zhen Wang, Side Hu, Julien Lescar, James P. Tam, Chuan-Fa Liu

**Affiliations:** School of Biological Sciences, Nanyang Technological University, Singapore 637551, Singapore; DINGPENG001@e.ntu.edu.sg (D.Z.); ZHEN002@e.ntu.edu.sg (Z.W.); side001@e.ntu.edu.sg (S.H.); julien@ntu.edu.sg (J.L.)

**Keywords:** biocatalysts, VyPAL2, protein labeling, protein cyclization, cell surface labeling

## Abstract

The last two decades have seen an increasing demand for new protein-modification methods from the biotech industry and biomedical research communities. Owing to their mild aqueous reaction conditions, enzymatic methods based on the use of peptide ligases are particularly desirable. In this regard, the recently discovered peptidyl Asx-specific ligases (PALs) have emerged as powerful biotechnological tools in recent years. However, as a new class of peptide ligases, their scope and application remain underexplored. Herein, we report the use of a new PAL, VyPAL2, for a diverse range of protein modifications. We successfully showed that VyPAL2 was an efficient biocatalyst for protein labelling, inter-protein ligation, and protein cyclization. The labelled or cyclized protein ligands remained functionally active in binding to their target receptors. We also demonstrated on-cell labelling of protein ligands pre-bound to cellular receptors and cell-surface engineering via modifying a covalently anchored peptide substrate pre-installed on cell-surface glycans. Together, these examples firmly establish Asx-specific ligases, such as VyPAL2, as the biocatalysts of the future for site-specific protein modification, with a myriad of applications in basic research and drug discovery.

## 1. Introduction

Peptide ligases are enzymes of relatively rare occurrences when compared to proteases, their ubiquitous counterparts. Unlike the hydrolytic proteases, peptide ligases catalyze the formation of new peptide bonds [1,2,3,4,5,6,7]. They perform ligation and cyclization reactions on peptides and proteins with exquisite site-specificity, and, generally, under physiological conditions [1,2,3,4,5,6,7]. Naturally-occurring peptide ligases are found in organisms such as bacteria and plants, serving functions such as the assembly of bacterial pili, and biosynthesis of cyclic peptides for host-defense [7,8,9,10,11,12]. Butelase-1, isolated from the tropical plant *Clitoria ternatea*, is the first plant-derived peptidyl Asx-specific ligase (PAL) [13]. It catalyzes peptide bond formation at Asn/Asp residues in two steps—the cysteinyl thiol at the enzyme’s active site first attacks an Asx-Xaa peptide bond in the acyl donor substrate to form an acyl-enzyme thioester intermediate, which then undergoes aminolysis by the N-terminal amino group of an incoming nucleophile peptide substrate [1]. Structurally, butelase-1 belongs to the same family as asparaginyl endopeptidases (AEPs) [14]. Many AEPs possess dual peptidase and ligase activities [15,16,17,18,19,20,21,22,23,24]. Butelase-1 is unique in that it is a pure PAL that does not have the typical hydrolytic activity of an AEP, at least in catalyzing peptide backbone cyclization reactions [13]. The catalytic efficiency of butelase-1 is far greater than previously known ligases, such as sortase A [1,13]. Recent reports have confirmed the versatility of butelase-1-mediated, as well as other PAL-mediated ligation, for protein and peptide modifications [13,20,21,22,23,24,25,26,27,28,29,30,31,32,33,34,35,36,37,38]. Examples include butelase-mediated labelling of live bacterial and mammalian cells at near physiological conditions [31,34]. In this regard, the mild enzymatic ligation approaches using a peptide ligase, such as butelase-1, provide a clear advantage over chemical ligation methods, particularly in situations where the protein substrates are in their native folded state.

Although many AEPs are shown to have a certain level of ligase activity at near neutral pH [15,16,17,18,19,20,21,22,23,24], the number of those that quality as pure PALs (i.e., those without the undesirable hydrolase activity) remains very limited [13,25]. In search for new PALs with similar enzymatic profiles as butelase-1, we have recently identified and characterized VyPAL2 from *Viola yedonesis* using bioinformatic and biochemical approaches [25]. As it has been demonstrated to use many peptide substrates, this newly found ligase has fast catalytic kinetics. Also, its recombinant form can be produced in high yield [25,35]. The discovery of VyPAL2 suggests that it is still possible to find highly active new PAL enzymes from plants. It also helped identify a set of the so-called major ligase activity determinants that can be used to design an aminolytic PAL from a hydrolytic AEP through rational engineering [36].

In addition to PALs, well-established peptide ligases include subtiligase [39,40,41,42,43] and Sortase A [44,45,46,47]. However, these peptide ligases have certain drawbacks which hinder their broad applications. For example, subtiligase can only use peptide C-terminal esters or thioesters as the acyl donor substrates [39,40,41,42,43]. The difficulty involved in preparing such substrates is a limiting factor for subtiligase-mediated ligation. Sortase A is a very slow enzyme which is needed in stoichiometric quantities as the substrate to ensure acceptable efficiency of the ligation reaction. It also requires a long recognition tag LPXTG, and, as such, its ligation products would bear a large extra “scar” at ligation junctions [44,45,46,47]. The ligation reactions catalyzed by the PALs are traceless, leaving only an asparagine residue in the products, which can be chosen from one of the Asn residues in the native protein sequence. Furthermore, because of the high efficiency of PALs, which are generally used in <1 mol% of their substrates, the ligation products can be easily isolated or often used directly without further extensive treatments. Finally, the substrates for PAL-mediated ligations are generally synthetic peptides or unmodified recombinant proteins, which can be conveniently prepared chemically, or expressed in large quantities.

**Scheme 1:**
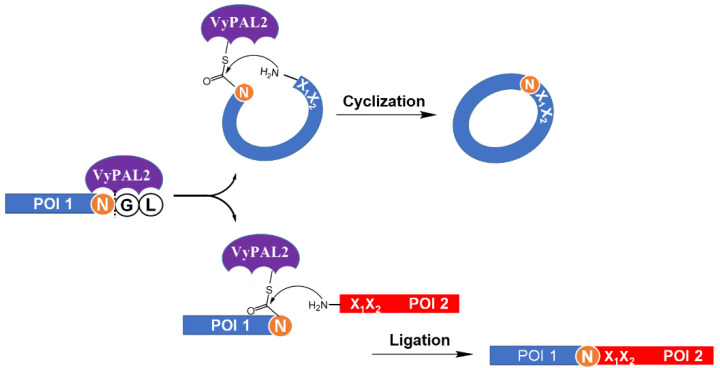
A general scheme of VyPAL2-mediated intramolecular and intermolecular ligation reactions. NGL (Asn-Gly-Leu) = a C-terminal recognition motif for VyPAL2. POI 1 and POI 2 = protein of interest 1 and 2.

**Scheme 2:**
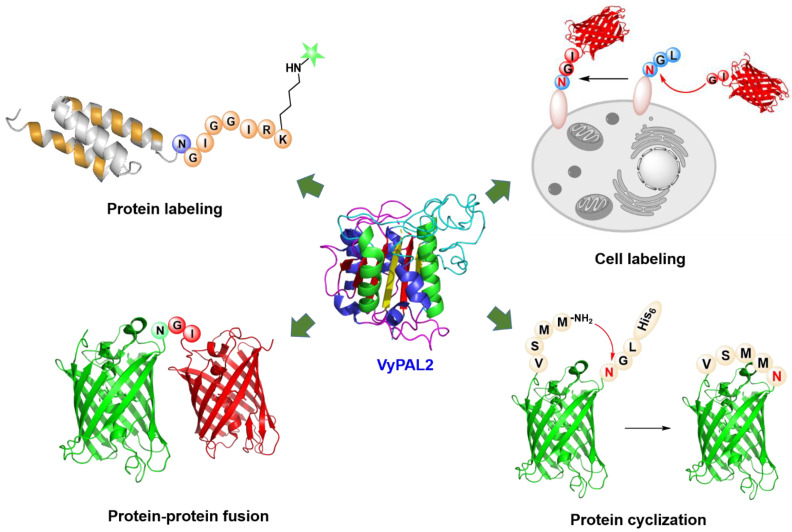
Examples of VyPAL2-mediated ligation (VML) demonstrated in this study.

In this study, we show that VyPAL2 is a highly efficient PAL enzyme for protein ligation and cyclization (Scheme 1). Using substrates designed to carry short recognition motifs of the ligase, we have demonstrated the versatility of VyPAL2 in a number of applications, including protein labeling, inter-protein ligation, and backbone circularization, as well as “in-situ” cell-surface modification following prior functionalization of the cell surface with an acyl donor substrate of the ligase (Scheme 2). The results obtained in the present study, together with those of previously published studies, point to the great promise of VyPAL2 in the development of protein biologics and engineered live cells for various applications in biotechnology and medicine, such as the treatment of human diseases.

## 2. Results and Discussion

### 2.1. Demonstration of VML for Protein Labeling and the Use of the Labelled Protein in Cell Imaging

#### 2.1.1. VyPAL2-Mediated Ligation for Protein C-Terminal Labeling

**Figure 1:**
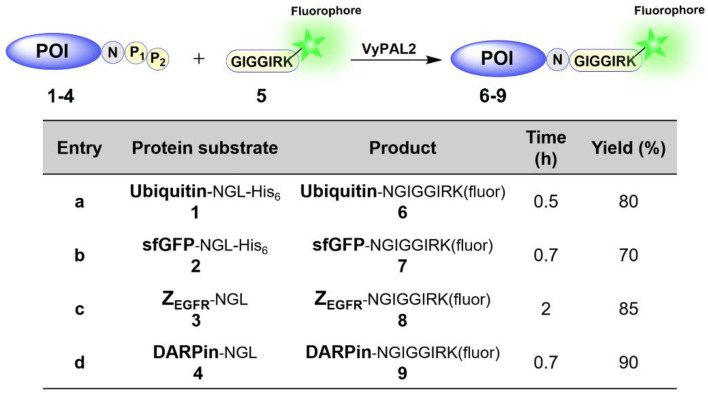
VyPAL2-mediated protein C-terminal labeling with a fluorescent peptide. See Supporting Information for detailed experimental conditions, procedures, and analytical data. All yields reported in this study are conversion yields based on HPLC analysis.

**Figure 2:**
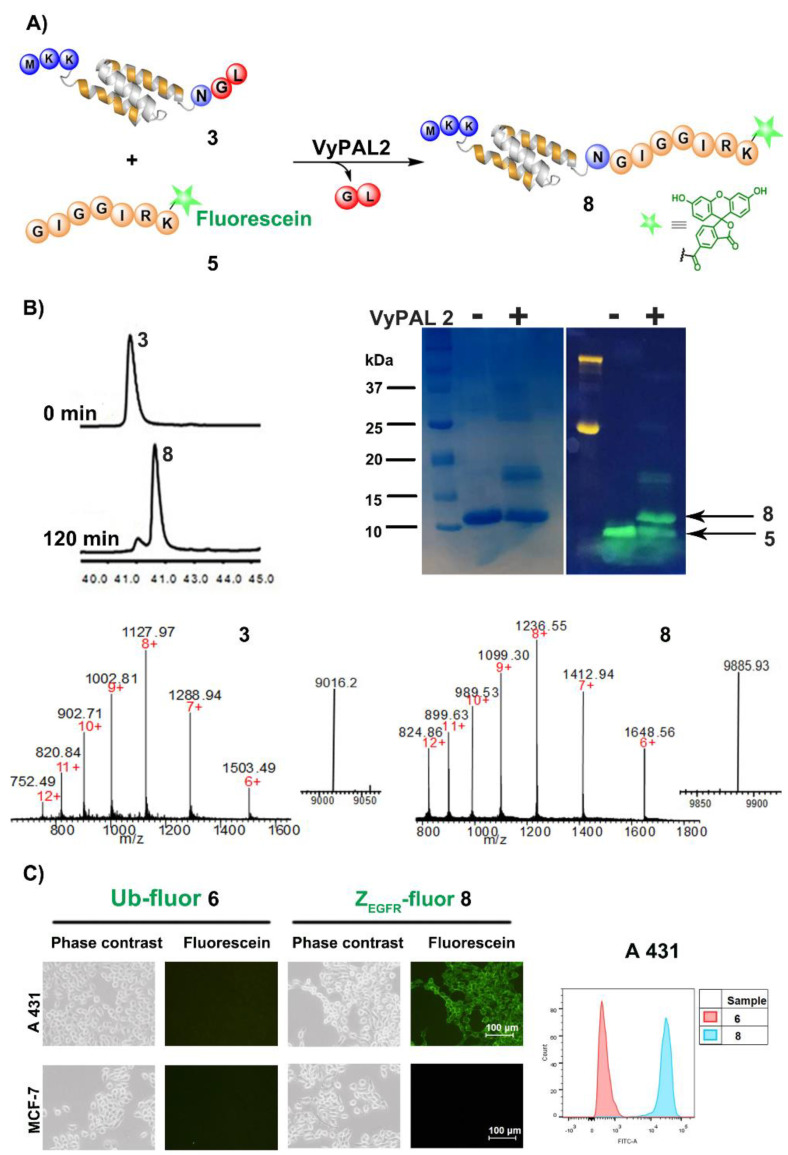
C-terminal labeling of Z_EGFR_ **3** by fluorescent peptide **5** for cell binding analysis. (**A**) The labelling reaction scheme. (**B**) Monitoring of the reaction by HPLC and SDS gel (upper panel; note: the band corresponding to peptide **5** on the Coomassie gel image was washed away by the destaining solution during the destaining step), and ESI-MS analysis of the labelling product (lower panel; **3**: Calcd. 9014.1, Obsvd. 9016.2; **8**: Calcd. 9883.5, Obsvd. 9885.9). (**C**) Fluorescent microscopy imaging and flow cytometry analysis. Fluorescence-labeled affibody **8** and ubiquitin **6** (Figure S2) were used to treat cancer cell lines MCF-7 (EGFR-low expressing) and A431 (EGFR-overexpressing), and the treated cells were subjected to flow cytometry analysis and fluorescent microscopy. As shown in (**C**), only **8** was able to bind on the EGFR+ A431 cells. As the negative control, **6** bound to neither MCF-7 nor A431 cells.

To show that VyPAL2 can ligate proteins as efficiently as model peptides [25,35], we first tested it for the labeling of several model proteins of various sizes: ubiquitin, a DARPin protein specific for the HER2 antigen [48], an affibody specific for EGFR (Z_EGFR_) [49], and sfGFP (Figure 1). These protein substrates were designed for C-terminal modification. To this end, all proteins were genetically modified with a C-terminal tag containing the tripeptide motif NGL to give Ub-NGL-His_6_ **1**, sfGFP-NGL-His_6_ **2**, Z_EGFR_-NGL **3**, and DARPin-NGL **4** (Figure 1). The C-ter His tag present in protein **1** and **2** also makes it straightforward to remove any unreacted protein substrate by a simple affinity chromatography filtration process [26,33]. VyPAL2-mediated ligation (VML) was conducted to label the engineered proteins with the fluorescent peptide **5** using a 1-to-5 (protein/peptide) ratio. In each reaction, 50–100 μM protein substrate, **1**–**4**, was reacted with 250–500 μM peptide **5** in the presence of 50 nM of VyPAL2 for 0.5–2 h at 37 °C. In all cases, a clean conversion was obtained, as verified by HPLC and MS analysis (Figure 2B and Figures S2–S4), and the reactions afforded the labeled protein products in good to excellent yields (Figure 1). These data confirm that VyPAL2 is a highly efficient asparaginyl peptide ligase useful for protein modification.

#### 2.1.2. Binding of Fluorescence-Labeled Affibody **8** to A431 Cells

As mentioned above, Z**_EGFR_** is an affibody that binds specifically to the human EGF receptor [49]. Fluorescence tagging makes it easy to detect the binding activity of **Z**_EGFR_ to EGFR-expressing cells. As such, affibody **8** and ubiquitin **6** were used to treat cancer cell lines MCF-7 (with very low expressing levels of EGFR) and A431 (EGFR-overexpressing) [50]. The treated cells were subjected to flow cytometry analysis and fluorescent microscopy imaging. As shown in Figure 2C, only **8** was able to bind on the EGFR+ A431 cells. As the negative control, **6** bound to neither MCF-7 nor A431 cells. Approaches allowing for site-specific modification of protein ligands with a detection tag hold great value in the study of biomolecular interactions and drug discovery research, as well as in imaging-based diagnosis and management of cancer and other diseases. The fast and specific labeling reactions of the proteins shown herein demonstrate the great potential of VyPAL2 for these applications.

### 2.2. VyPAL-Mediated Inter-Protein Ligation

**Figure 3:**
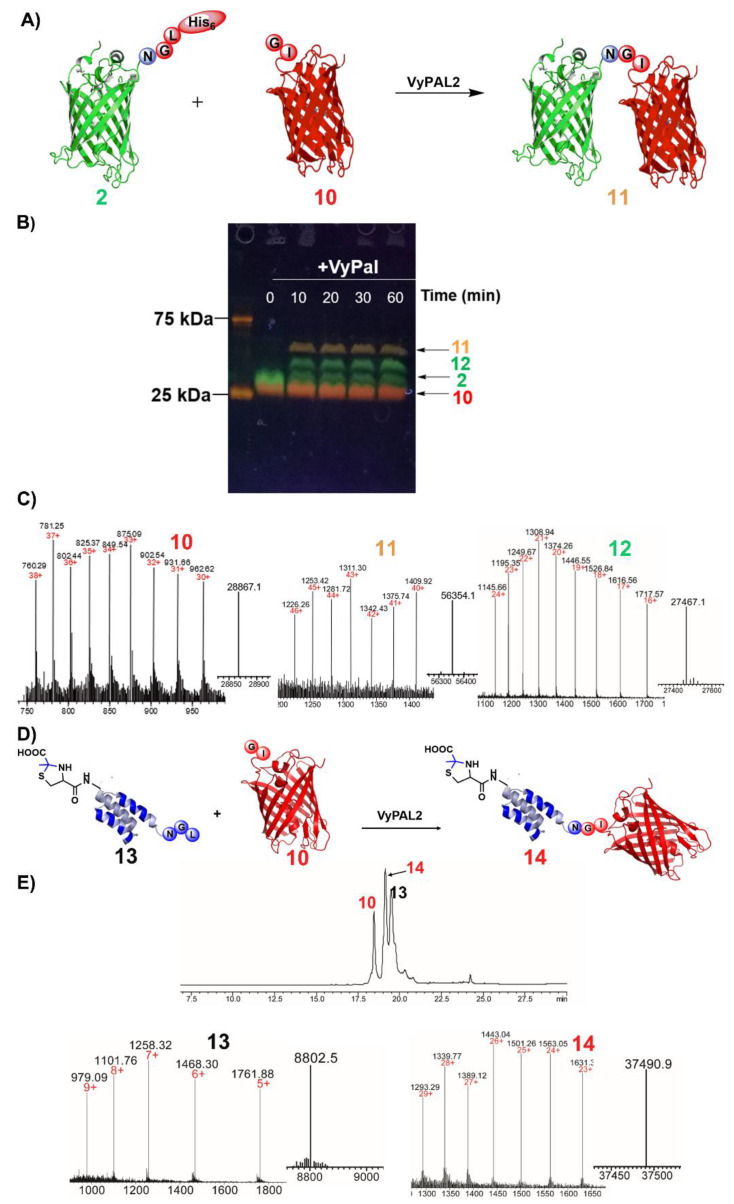
VyPAL2-mediated inter-protein ligation. (**A**) Schematic illustration of ligation reaction between the green fluorescent protein sfGFP engineered with a C-terminal tripeptide “NGL” **2** and the red fluorescent protein GI-mCherry **10**. (**B**) Fluorescent gel analysis of the ligation reaction between sfGFP-NGL **2** and GI-mCherry **10** catalyzed by VyPAL2. Aliquots of the reaction mixture were taken out and frozen at different time points, and, at the end, subjected to SDS-page gel electrophoresis. (**C**) ESI-MS characterization of GI-mCherry **10**, inter-protein ligation product **11**, and the cyclic by-product **12** (**10**: calcd mass 28,860.4 and obsvd mass 28867.1; **11**: calcd mass 56,350.5 and obsvd mass 56,354.1; **12**: calcd mass 27,474.9 and obsvd mass 27,467.1). (**D**) Schematic illustration of the ligation reaction between the EGFR-targeting affibody Z_EGFR_ tagged with the C-terminal tripeptide “NGL” **13** and the red fluorescent protein GI-mCherry **10**. (**E**) HPLC and ESI-MS analysis of the ligation reaction between **13** and **10** catalyzed by VyPAL2. **13** and **10** were mixed at a 2:1 ratio. The reaction was kept in room temperature for 30 min, then subjected to the HPLC analysis. ESI-MS characterization of Z_EGFR_-NGL **13** and the inter-protein ligation product **14** (**13**: calcd mass 8799.7 and obsvd mass 8802.5; **14**: calcd mass 37,481.2 and obsvd mass 37,490.9).

Next, we asked whether VyPAL2 could be used for inter-protein ligation. So far, there has been no reported examples of ligating large proteins with the PAL enzymes. To test VyPAL2 in a protein-protein ligation, sfGFP-NGL-His_6_ **2** was first used as the acyl donor substrate. The nucleophile protein substrate was GI-mCherry **10**, which has the Gly-Ile dipeptide at the N-terminus. The ligation reaction was performed using 15 μM sfGFP-NGL **2** and 30 μM GI -mCherry **10** and 100 nM VyPAL in phosphate buffer (pH 7.4) at 37 °C. The reaction was monitored at 0, 10, 20, 30, and 60 min by subjecting the reaction mixture to fluorescent PAGE gel analysis (Figure 3B). The ligation product **11** of the green sfGFP **2** with red mCherry **10** was of yellow color (Figure 3B). As seen from Figure 3B, the yield of the ligation reaction reached about 30% at 10 min, but the yield did not seem to improve with prolonged reaction time. The ligation product was also isolated using HPLC, and its identity was confirmed by ESI-MS (Figure 3C). It was found that the band between the ligation product and the starting GFP substrate **2** corresponded to the backbone cyclized form of sfGFP, which was formed as a result of intramolecular ligation between the Asn residue on C-terminal tail, and the Met residue at the N-terminus, even though the N-terminal Met-Met dipeptide of **2** is not a favored nucleophile substrate for VyPAL2. This band seemed to become slightly more intensive over time. Therefore, this competing cyclization reaction is one of the reasons for the relatively low yield of the inter-protein ligation between the two large fluorescent proteins **2** and **10**. The close proximity of the N-terminal and C-terminal ends of sfGFP makes it particularly prone to the cyclization reaction. The other reasons are likely the inherent steric hindrance and slow diffusion kinetics of the large nucleophile protein **10**, since the same substrate **2** was able to ligate with the small synthetic peptide **5** in a very good yield, as seen in Figure 1 above. Next, we applied the method to ligate the affibody with mCherry. The intrinsic red fluorescent signal of mCherry would be very useful in cell imaging and diagnostic applications. To block the N terminus from being engaged in intramolecular self-cyclization, the affibody was engineered with a Cys at the second residue following the N-terminal Met. The Met residue was removed during the expression process in E. coli. We found that the purified affibody was already capped at the exposed N-terminal Cys by pyruvate, a common cellular metabolite, forming a thiazolidine. This essentially prevents the protein from undergoing N-to-C cyclization during VML. We used Thz-Z_EGFR_-NGL **13** in excess of **10** for the inter-protein ligation reaction. So, Thz-Z_EGFR_-NGL **13** (80 μM) and GI-mCherry **10** (40 μM) were mixed with 200 nM VyPAL2 in phosphate buffer (pH 7.4) at 37 °C. The reaction was monitored at 0, 5, 10, 15, and 30 min by subjecting the reaction mixture to fluorescent PAGE gel analysis (Figure S5). After ligating with mCherry, the product **14** was visible as a red fluorescent band (Figure S5). As seen from Figure S5, the yield of the ligation reaction was estimated to be about 60% on the basis of **10**. The reaction mixture at 30 min was analyzed by HPLC, which also indicated a yield of about 60% of the affibody-mCherry fusion protein **14,** and the isolated ligation product was checked by ESI-MS (Figure 3E).

### 2.3. VML for Protein Cyclization

#### 2.3.1. PAL-Mediated Affibody Z_EGFR_ and DARPin Cyclization

**Figure 4:**
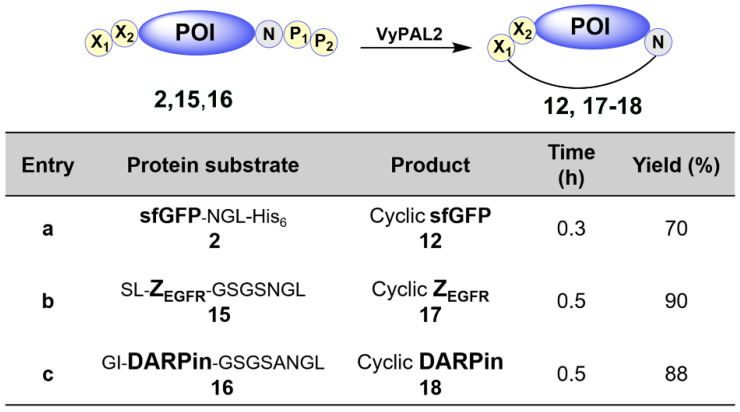
VyPAL2-mediated protein macrocyclization.

**Figure 5:**
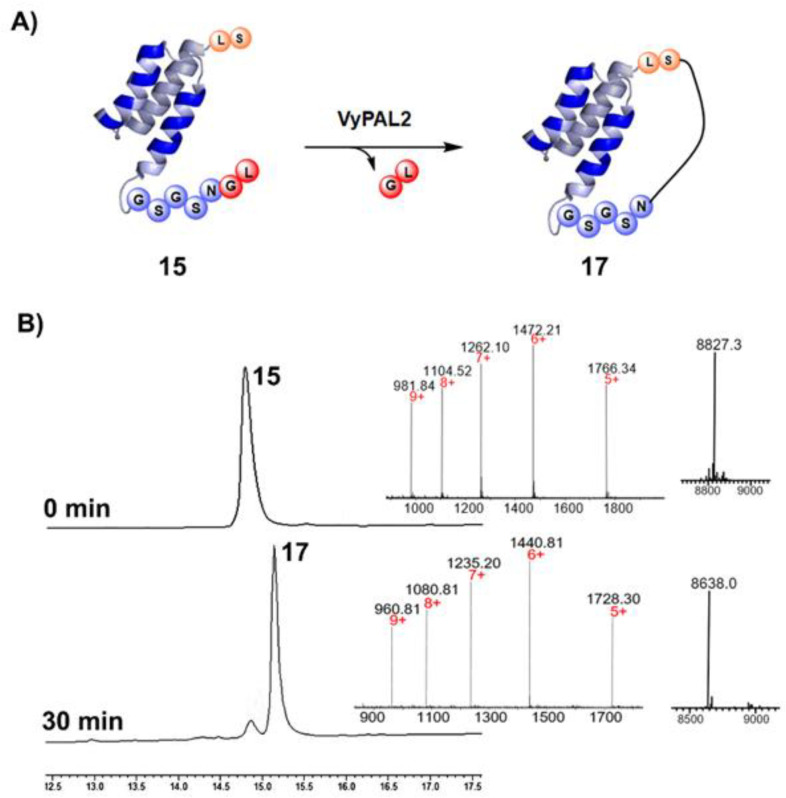
VyPAL-mediated macrocyclization of an EGFR-targeting affibody **15**. (**A**) Scheme of VyPAL-mediated Z_EGFR_ **15** cyclization; (**B**) characterization of VyPAL-mediated Z_EGFR_ **15** cyclization by HPLC and ESI-MS analysis. Z_EGFR_ **15**: calcd mass 8826.8 and obsvd m/z [M+H]^+^ 8827.3; product **17**: calcd mass 8638.8 and obsvd m/z [M+H]^+^ 8638.0.

The transpeptidase activity of many AEPs is known to be responsible for the biosynthesis of naturally occurring cyclopeptides in plants. Indeed, butelase-1 and a few PALs have been found to be particularly efficient in catalyzing the cyclization reaction of synthetic peptides and proteins [1,13,19,20,28,29,33,35]. The cyclization of therapeutic proteins would help develop a new generation of biologics for disease treatment [51]. Compared with their linear counterparts, the cyclic proteins usually exhibit improved pharmacokinetic properties due to their increased proteolytic stability and enhanced thermo stability [52]. In this study, we performed VyPAL2-mediated cyclization on three proteins (Figure 4). For a typical cyclization reaction, we mixed 50 μM of protein substrate with 100 nM of VyPAL2 in pH 7.4 for 0.3–0.5 h. Since we observed significant cyclization of **2** in the previous inter-protein ligation experiments, it was used directly for the cyclization reaction. Indeed, in the absence of another nucleophile, the protein substrate **2** underwent exclusive cyclization, and 70% of the cyclic sfGFP **12** was formed in 0.3 h. The other two proteins used were the affibody **15** and DARPin **16**. For these two proteins, a short spacer of 4 or 5 amino acid residues, respectively, was added before the NGL motif to facilitate the intramolecular ligation, which afforded the cyclic products **17** and **18** in excellent yields (Figure 4). The reactions were monitored by HPLC, and the products were characterized by ESI-MS (Figure 5, Figures S6 and S7).

#### 2.3.2. Binding of Cyclic Affibody and Cyclic DARPin to Their Target Receptors

**Figure 6:**
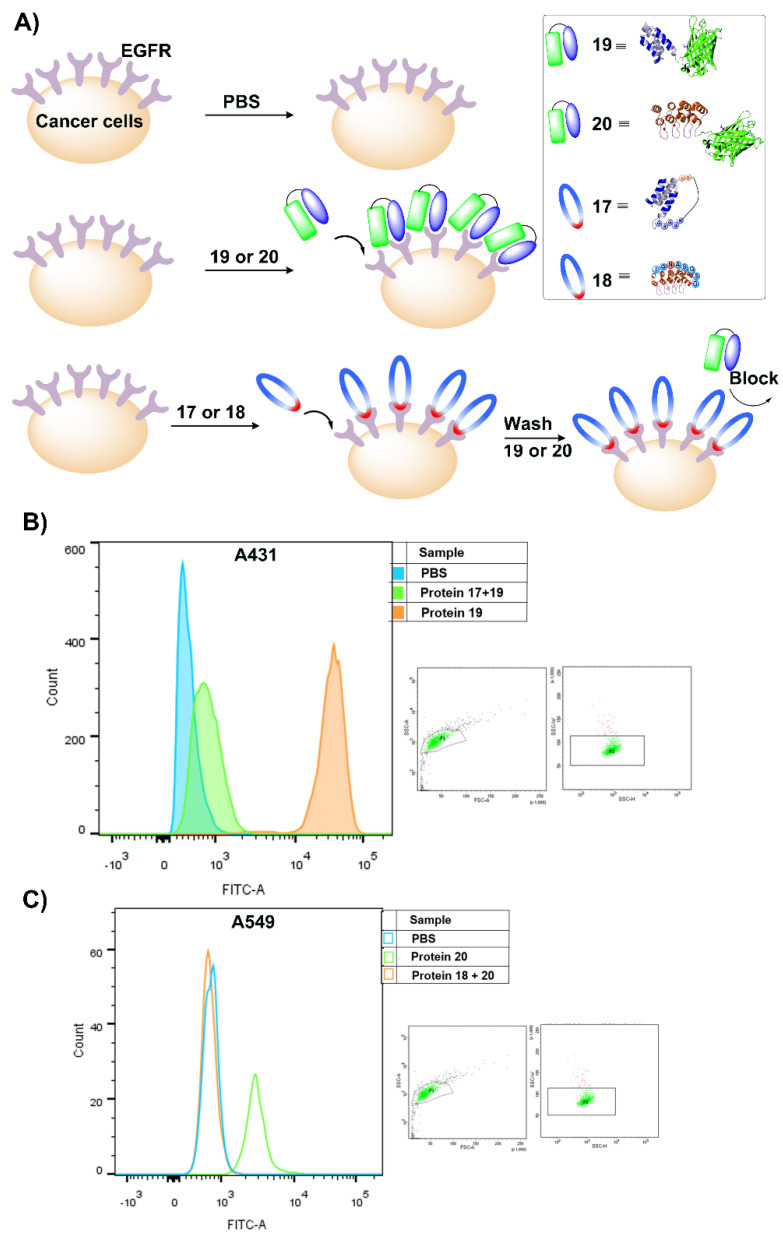
Receptor competitive binding assay to evaluate cyclic affibody Z_EGFR_ **17** and DARPin **18** binding to the target receptors. (**A**) Schematic representation of the receptor competitive binding assay using the cyclic affibody as the example; (**B**) flow cytometry analysis of cyclic affibody **17** binding to the EGFR receptor; (**C**) flow cytometry analysis of cyclic DARPin **18** binding to EGFR receptor.

A competitive receptor binding assay was performed using the cyclic Z_EGFR_ **17** against the sfGFP-tagged Z_EGFR_ **19** on A431 cells, which overexpresses EGFR or cyclic DARPin **18** against the sfGFP-tagged DARPin **20** on A549 cells, which overexpresses HER2 (Figure 6A). The two GFP fusion proteins, Z_EGFR_-sfGFP **19** and DARPin-sfGFP **20**, were prepared recombinantly in *E. coli*. Cells were first treated with 100 nM of cyclic Z_EGFR_ **17** or DARPin **18**, and after washing away unbound **15** or **16** with 20 nM of Z_EGFR_-sfGFP **19** or DARPin-sfGFP **20**. As a positive control, cells were treated only with Z_EGFR_-sfGFP **19** or DARPin-sfGFP **20**. The samples were subjected to flow cytometry analysis after washing with PBS three times. The positive control group showed strong FITC signal shift in flow cytometry analysis, whereas cells pre-saturated with cyclic **17** and **18** showed minimum signal shift (Figure 6B,C). As such, this FACS analysis indicated that the binding capability of cyclic Z_EGFR_ **17** and cyclic DARPin **18** remained intact.

### 2.4. VyPAL2-Mediated Ligation on Cell Surface

#### 2.4.1. VML for the Labelling of Affibody and DARPin Pre-Bound on Cell Surface

**Figure 7:**
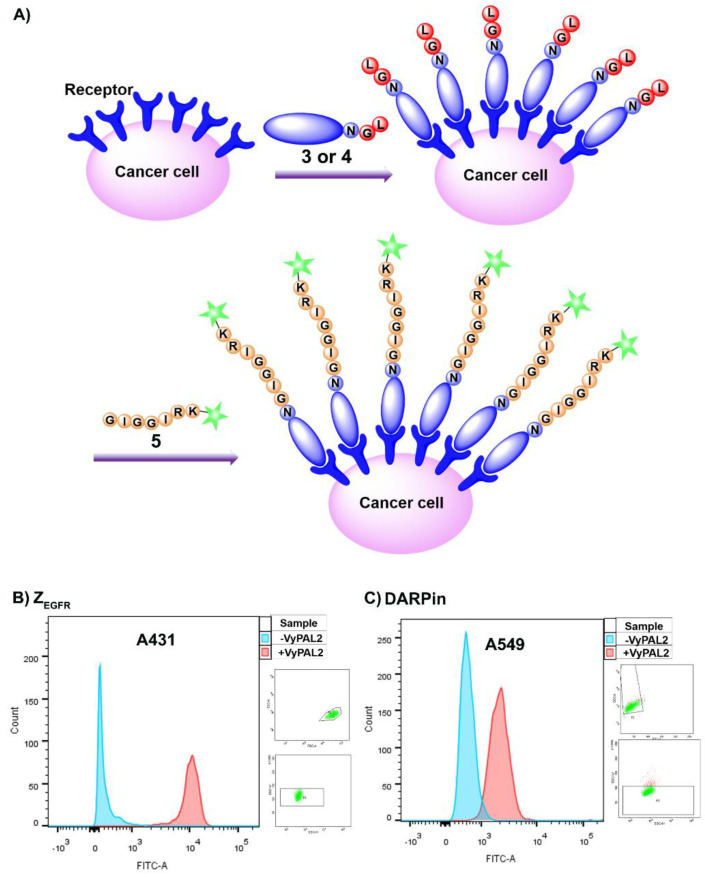
Labeling of cell surface-bound affibody **3** and DARPin **4**. (**A**) Reaction scheme of labelling protein ligands which are pre-bound to cell surface receptors; (**B**) flow cytometry characterization of A431 cells with pre-bound Z_EGFR_ **3** on cell surface after incubation with **5** in the presence or absence of VyPAL2; (**C**) flow cytometry characterization of A549 cells with pre-bound DARPin **4** on cell surface after incubation with **5** in the presence or absence of VyPAL2.

As previously shown, VML was highly effective for the C-terminal labelling of Z_EGFR_ and Her2-specific DARPin. We wanted to test the same labelling conditions on the two proteins that were pre-bound on cell surface receptors. To perform the experiment, 30 ul of a stock solution of affibody **3** or DARPin **4** (5 mg/mL) was added to A431 or A549 cells in 150 ul PBS, and, after incubation for 30 min, the cells were washed three times with PBS to remove the unbounded proteins. Next, peptide **5** (50 μM) was added. Finally, the cells were separated into two groups, one was treated with 200 nM VyPAL2, and one, as the blank control, was treated with PBS. The resulting cells were analyzed by flow cytometry, and the data are presented in Figure 7 (Figure 7B). From that, we can conclude that VyPAL2 was efficient in catalyzing the labelling reaction of a cell surface-bound protein. As another control, when the cells were not pre-incubated with affibody **3** or DARPin **4**, labeling with peptide **5** by VyPAL2 did not give any fluorescence-labeled cells (data not shown).

#### 2.4.2. VML for Labeling a Cell-Surface Glycan Anchored Peptide Substrate Preinstalled via Oxime Conjugation

**Figure 8:**
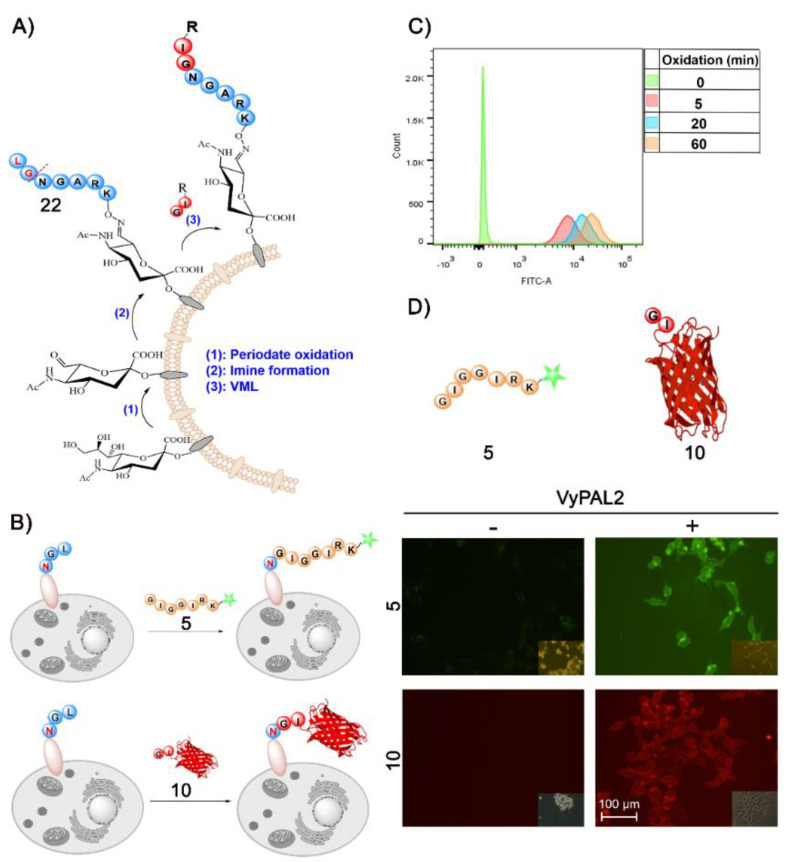
A cell surface modification strategy based on consecutive oxime ligation and VML. (**A**) Schematic illustration of cell surface modification in three steps: (1) sodium periodate oxidation of sialic acid on cell-surface glycans (by 10 mM sodium periodate in PBS at 0 °C for 5 min); (2) imine formation (oxime conjugation) by treating A431 cells with 1 mM of the aminooxyl-peptide **22** to anchor the PAL substrate onto the cell surface. (3) VML was performed by treating the cells with 0.5 mM of GI-peptide or GI-protein for 30 min. (**B**) Schematic illustration of VML for the labeling of pre-installed cell-surface VyPAL2 substrate with the fluorescein-peptide **5** (upper panel) or mCherry **10** (low panel). (**C**) Flowcytometry analysis of cells after labeling with fluorescein-peptide **5** using the three-step scheme. Time course study of sodium periodate treatment indicated the efficiency of a 5-min treatment. (**D**) Fluorescent microscopy imaging analysis of cells labeled with fluorescein-peptide **5** and mCherry **10**. Negative control was done by treating A431 cells with **5** or **10** in the absence of VyPAL2.

The above experiment has demonstrated that VML can be used to label VyPAL2 protein substrates non-covalently associated with cell surface through ligand–receptor interactions. In the following, we explored the possibility of using VML to label VyPAL2 substrates that are covalently bonded to the cell surface. In this strategy, the cells were first subjected to mild periodate oxidation by treatment with 10 mM sodium periodate on ice for 5 min, which generated aldehyde groups through oxidizing the sialic acid residues on cell-surface glycans. Next, a peptide containing the –NGL tripeptide motif at the C terminus as the VyPAL2 substrate and an N-terminal aminooxyl functional group was used to react with the surface aldehydes through oxime formation, which decorated the cell surface with the VyPAL2 substrate. We verified this reaction by performing a parallel experiment in which a synthetic peptide, peptide **21,** with the same amino acid sequence, but containing a biotin tag on the lysine residue, was used for oxime conjugation. Flowcytometry analysis using the avidin-FITC probe confirmed the presence of the biotin tag on the cell surface (Figure S9). We then proceeded with the oxime conjugation using peptide **22 (**H_2_NOCH_2_CO-**KRAGNGL)**. Following this, VML was carried out by the addition of 0.5 mM of the GI- containing fluorescein-peptide **5**, or mCherry **10** and 100 nM of VyPAL2 to the cells in PBS buffer (Figure 8A). After incubation for 30 min, the cells were washed with PBS three times, before subjecting to flowcytometry analysis. Our data showed that a 5-min periodate oxidation of the cells was able to generate enough aldehyde groups to decorate the cells with a sufficient amount of the VyPAL2 peptide substrate, which could then be labeled with the peptide **5** or protein nucleophile **10** via VML (Figure 8B). Fluorescent microscopy analysis showed that both the fluorescein-peptide and mCherry were conjugated to the cell surface, as seen in intense green and red fluorescence images of the labelled cells (Figure 8B,C). These results show that this oxime-VML consecutive ligation strategy is effective for labeling cell surfaces with various functional moieties. Such a strategy may find useful applications in basic biomedical research and translational medicine, such as the study of trafficking and other biological processes of the cell surface components, and the development of cell-based therapies.

## 3. Materials and Methods

All chemicals and amino acids were purchased from Sigma-Aldrich (Merck Singapore) and Chemimpex (Wood Dale, IL, USA) unless otherwise indicated. All solvents and reagents were used as received without further purification. VyPAL2 was prepared in-house, as previously reported.

Materials used in the study include phosphate saline buffer (Gibco^TM^, ThermoFisher Scientific, Singapore), nitrilotriacetic acid-nickel (Ni-NTA, Biobasic, Singapore), Dulbecco’s Modified Eagle Medium (DMEM, Gibco), fetal bovine serum (FBS, Gibco), ethylenediaminetetraacetic acid (EDTA), 4-Methylbenzhydrylamine resin (MBHA resin, Chemimpex), Fluorenylmethyloxycarbonyl-amino acids (Fmoc-amino acids), tert-butyloxycarbonyl-amino acids (Boc-amino acids), trifluoroacetic acid (TFA), triisopropylsilane (TIS), dimethylformamide (DMF), dichloromethane (DCM), benzotriazol-1-yl-oxytripyrrolidinophosphonium hexafluorophosphate (PyBOP, Chemimpex), N,N-Diisopropyl- ethylamine (DIPEA, Chemimpex), isopropyl β-D-1-thiogalactopyranoside (IPTG, Biobasic, Singapore), fluorescein (Sigma), LB broth (Biobasic, Singapore).

### 3.1. HPLC

Analytical RP-HPLC was run on a SHIMADZU (Prominence LC-20AT) instrument using an analytical column (Grace Vydac “Protein C4”, 250 × 4.6 mm, 5 µm particle size) at a flow rate of 1.0 mL/min. Analytical HPLC elution was monitored by UV absorption at 214 nm and 254 nm. Semi-preparative RP-HPLC was run on a SHIMADZU (Prominence LC-20AT) instrument using a semi preparative column (Grace Vydac “Protein C4”, 250 × 10 mm, 10 µm particle size) at a flow rate of 2.5 mL/min. Both analytical and semi-preparative HPLC were run at room temperature using a gradient of solvent B in solvent A. Solvent B was 90% acetonitrile in water (0.040% TFA), and solvent A was water (0.045% TFA). Both solvents were filtered through 0.22 µm filter paper, and sonicated for 30 min before use.

### 3.2. Mass Spectrometry

ESI mass spectrum data of synthetic peptides and proteins were obtained from a Thermo Finnigan LCQ DECA XP MAX (ESI ion source, positive mode). The software of MagTran 1.03 and ESIProt 1.0 was used for the data deconvolution.

### 3.3. Molecular Cloning and Protein Expression

Genes encoding the desired protein sequences (see Supporting Information) were cloned into pETDuet vector, and the plasmids were then transformed into *E. coli* BL21 (DE3) competent cells by the standard 90 s heat shock protocol. The bacterial colonies were then picked up and transferred to liquid LB medium in a culturing flask. The flask was shaken in the incubator at 37 °C until the OD reached 0.6–0.8, followed by induction with 1 mM of IPTG at 37 °C in 4–8 h for protein expression. Cells were harvested and lysed by sonication in lysis buffer containing 50 mM sodium phosphate and 500 mM NaCl (pH = 8.0). After centrifugation, the supernatant was loaded on a column of Ni-NTA beads, and incubated at 4 °C for 1 h. The beads were washed three times with the lysis buffer, and the protein was subsequently eluted with lysis buffer containing 250 mM imidazole. The purified protein was dialyzed in phosphate buffer (pH = 6.5) overnight, and stored in the freezer at −20 °C.

### 3.4. Cell Culture and Imaging

Cells were maintained in 10% FBS in DMEM (high glucose) at 37 °C in an incubator under 5% CO_2_. For passaging, cells were first washed three times with trypsin-EDTA (0.25%) to detach the cells from tissue culture plates. Then, a 3-time volume of complete DMEM medium was added to neutralize trypsin activity. Cells were grown until 40–60% confluency. Peptides or proteins in complete medium were applied to the cells, and incubated for 30 min at 37 °C. Washing was done three times with PBS, and cells were subsequently subjected to microscopy analysis.

### 3.5. Flow Cytometry (FACS) Analysis

Prior to FACS analysis, cells were maintained in 6-well plates in DMEM medium supplemented with 10% FBS for 3 days. Then, the cells were detached from the plates using 1 mL of trypsin-EDTA (0.25%) for 3–5 min under 37 °C. Then, the complete DMEM medium was added in 3 mL to neutralize the trypsin digestion. After that, cells were collected into 15 mL falcon tubes, and centrifuged at 2000× *g* for 5 min. The supernatants were removed, and cells were washed with PBS three times. The proteins were added to the cells for 30–60 min in room temperature. Then, cells were washed again with PBS three times before subjecting to FACS analysis. FACS was conducted using FITC gate as *X*-axis, and Histogram as *Y*-axis. Finally, Flowjo was used to analyze the data.

### 3.6. Solid Phase Peptide Synthesis (SPPS)

The peptides used in this study (peptide **5**, **21,** and **22**) were synthesized as C-terminal amide using Rink amide MBHA resin by standard Fmoc chemistry. Before use, the resin was pre-swelled in DMF for 20 min. Before the first coupling, an Fmoc deprotection procedure was performed using 20% piperidine in dimethylformamide (DMF) for 30 min. The resin was then washed with DMF, DCM, and DMF successively. For the coupling reactions, 3 eq. of Fmoc-AA-OH and 3 eq. of PyBOP were first dissolved in DMF/DCM (1:1). The mixture was added to the resin, followed by the addition of 6 eq. of DIEA. Coupling reactions were carried out for 60 to 90 min. Coupling efficiency was examined by ninhydrin test. For peptide **5**, after sequence assembly on the solid phase, the Boc group on the C-ter lysine side chain amine was removed with 1 M HCl in DCM for 30 min, then 5(6)-carboxyfluorescein was coupled to the side-chain free amine using PyBOP as the coupling reagent. For peptides **21** and **22**, after the coupling and deprotection of the final amino acid, Fmoc-Lys(Biotin)-OH for **21** or Fmoc-Lys(Boc)-OH for **22**, (Boc-aminooxy)-acetic acid was coupled. Thus, a Boc-protected aminoxyl group was introduced at the N-termini of the peptides. All the peptides were cleaved from the resin with a cocktail containing 95% TFA, 2.5% water, and 2.5% TIS for 2 h. After precipitation with cold diethyl ether, the crude peptides were purified using HPLC. The desired peptide was obtained in the powder form after lyophilization. The peptides were characterized by electrospray ionization mass spectrometry.

Peptides prepared in the study:

Peptide **5**: **GIGGIRK**(Fluorescein); 1057.65 (observed), 1057.35 (calculated).

Peptide **21**: H_2_NOCH_2_CO-**K**(Biotin)**RAGNGL**, 1013.67 (observed), 1014.20 (calculated).

Peptide **22**: H_2_NOCH_2_CO-**KRAGNGL**, 787.68 (observed), 787.89 (calculated).

## 4. Conclusions

In this study, we have demonstrated VyPAL2 as an efficient and versatile biocatalyst for protein modification reactions. First, highly effective protein labeling was demonstrated on substrates engineered to have a C-terminal asparaginyl tripeptide recognition motif. Second, inter-protein ligation was also successfully implemented using VML to ligate sfGFP-NGL or Z_EGFR_-NGL with GI-mCherry. Third, VyPAL2-mediated protein backbone macrocyclization was readily affected with protein substrates containing both the C-terminal tripeptide motif and a suitable N-terminal dipeptide nucleophile. The modified protein ligands, labeled with a fluorescent peptide or cyclized, showed intact binding ability to their receptors. Lastly, the labeling reaction was adopted to modifying substrates either non-covalently bound or covalently bonded to cell-surface components. The strategies developed in this study point to the great promise of VyPAL2 as an excellent bio-manufacturing tool for preparing complex protein- and cell-derived bioconjugates containing various functionalities for use in biomedical research and biotechnology. Therefore, the methodologies described in this study may pave a new way to the development of next-generation biologics for the diagnosis, prevention, and treatment of human diseases.

## Data Availability

Not applicable.

## Supplementary Materials

The following is available online at www.mdpi.com/article/10.3390/ijms23010458/s1.
