# Supplementary material for: Vypal2: A Versatile Peptide Ligase for Precision Tailoring of Proteins
Source: Int J Mol Sci. 2021 Dec 31;23(1):458. doi: 10.3390/ijms23010458 (PMC8745061; doi:10.3390/ijms23010458)
Supplement: Supplementary file 1 [file ijms-23-00458-s001.zip › ijms-1519564-supplementary.pdf]

VyPAL2: a versatile peptide ligase for precision tailoring of proteins

Dingpeng Zhang,[†] Zhen Wang,[†] Side Hu, Julien Lescar, James P. Tam*, and Chuan-Fa Liu*[†]

[†]School of Biological Sciences, Nanyang Technological University, 60 Nanyang Drive, Singapore 637551, Singapore

Table of Contents

a) **Protein amino acid sequences**
b) **VyPAL2-mediated ubiquitin labeling**
c) **VyPAL2-mediated sfGFP labeling**
d) **VyPAL2-mediated DARPin labeling**
e) **VyPAL2-mediated affibody and mCherry inter-protein ligation**
f) **VyPAL2-mediated sfGFP macrocyclization**
g) **VyPAL2-mediated DARPin macrocyclization**

a. Protein amino acid sequences

The optimized DNA sequences of DARPin and Z_{EGFR} wrere synthesized by Genscript. The following are the amino acid sequences of all the recombinant proteins used in this study.

Ub-NGL-His_6 **1**

MKKQIFVKTLTGKTITLEVEPSDTIENVKAKIQDKEGIPPDQQRLIFAGKQLEDGRTLSDYNIQK
ESTLH LVLRLRGGNGLHHHHHH

sfGFP-NGL-His_6 **2**

MMSVSKGEELFTGVVPILVELDGDVNGHKFSVRGEGEGDATNGKLTLKFICTTGKLPVPWPT
LVTTLTYGVQCFSRYPDHMKRHDFFKSAMPEGYVQERTISFKDDGTYKTRAEVKFEGDTLV
NRIELKGIDFKEDGNILGHKLEYNFNSHNVYITADKQKNGIKANFKIRHNVEDGSVQLADHYQ
QNTPIGDGPVLLPDNHYLSTQSVLSKDPNEKRDHMVLLEFVTAAGITHGMDELYKGSGSNGL
HHHHHH

Z_{EGFR}-NGL **3**

MKKGSSHHHHHHLQVDNKFNKEMWAAWEEIRNLPNLNGWQMTAFIASLVDDPSQSANLLA
EAKKLNDAQAPKVDGSGSNGL

DARPin-NGL **4**

MKKGGHHHHHHSSGGTENLYFQSDLGKKLLEAARAGQDDEVRILMANGADVNAKDFYGITP
LHLAAAYGHLEIVEVLLKHGADVNAHDWNGWTPLHLAAKYGHLEIVEVLLKHGADVNAIDNA
GKTPLHLAAAHGHLEIVEVLLKYGADVNAQDKFGKTPFDLAIDNGNEDIAEVLQKAAKLGSGN
GL

GI-mCherry **10**

GIGSSHHHHHHSSGLVPRGSSMVSKGEDDNMAIIKEFMRFKVHMEGSVNGHEFEIEGEGEG
RPYEGTQTAKLKVTKGGPLPFAWDILSPQFMYGSKAYVKHPADIPDYLKLSFPEGFKWERV
MNFEDGGVVTVTQDSSLQDGEFIYKVKLRGTNFPSDGPVMQKKTMGWEASSERMYPEDG
ALKGEIKQRLKLKDGGHYDAEVKTTYKAKKPVQLPGAYNVNIKLDITSHNEDYTIVEQYERAE
GRHSTGGMDELYK

CG- Z_{EGFR}-NGL **13**

CGSSHHHHHHLQVDNKFNKEMWAAWEEIRNLPNLNGWQMTAFIASLVDDPSQSANLLAEA
KKLNDAQAPKVDGSGSNGL

SL-Z_{EGFR}-NGL **15**
SLGSSHHHHHHLQVDNKFNKEMWAAWEEIRNLPNLNGWQMTAFIASLVDDPSQSANLLAE
AKKLNDAQAPKVDGSGSNGL

GI-DARPin-NGL **16**

GISGHHHHHSSGGTENGLYFQSDLGKKLLEAARAGQDDEVRILMANGADVNAKDFYGITPL
HLAAAYGHLEIVEVLLKHGADVNAHDWNGWTPLHLAAKYGHLEIVEVLLKHGADVNAIDNAG
KTPLHLAAAHGHLEIVEVLLKYGADVNAQDKFGKTPFDLAIDNGNEDIAEVLQKAANKLGSGS
ANGL

Z_{EGFR}-sfGFP **19**

SIGSSHHHHHHLQVDNKFNKEMWAAWEEIRNLPNLNGWQMTAFIASLVDDPSQSANLLAEA
KKLNDAQAPKVDGGGGSGGVSKGEELFTGVVPILVELDGDVNGHKFSVRGEGEGDATNGK
LTLKFICTTGKLPVPWPTLVTTLTYGVQCFSRYPDHMKRHDFFKSAMPEGYVQERTISFKDD
GTYKTRAEVKFEGDTLVNRIELKGIDFKEDGNILGHKLEYNFNSHNVYITADKQKNGIKANFKI
RHNVEDGSVQLADHYQQNTPIGDGPVLLPDNHYLSTQSVLSKDPNEKRDHMVLLEFVTAAGI
THGMDELYKGSGSNGLHHHHHH

DARPin-sfGFP **20**

MGSSHHHHHHSSGGTENLYFQSDLGKKLLEAARAGQDDEVRILMANGADVNAKDFYGITPL
HLAAAYGHLEIVEVLLKHGADVNAHDWNGWTPLHLAAKYGHLEIVEVLLKHGADVNAIDNAG
KTPLHLAAAHGHLEIVEVLLKYGADVNAQDKFGKTPFDLAIDNGNEDIAEVLQKAAKLGSGSS
KGEELFTGVVPILVELDGDVNGHKFSVRGEGEGDATNGKLTLKFICTTGKLPVPWPTLVTTLT
YGVQCFSRYPDHMKRHDFFKSAMPEGYVQERTISFKDDGTYKTRAEVKFEGDTLVNRIELK
GIDFKEDGNILGHKLEYNFNSHNVYITADKQKNGIKANFKIRHNVEDGSVQLADHYQQNTPIG
DGPVLLPDNHYLSTQSVLSKDPNEKRDHMVLLEFVTAAGITHGMDELYKGSGSNGLHHHHH
H

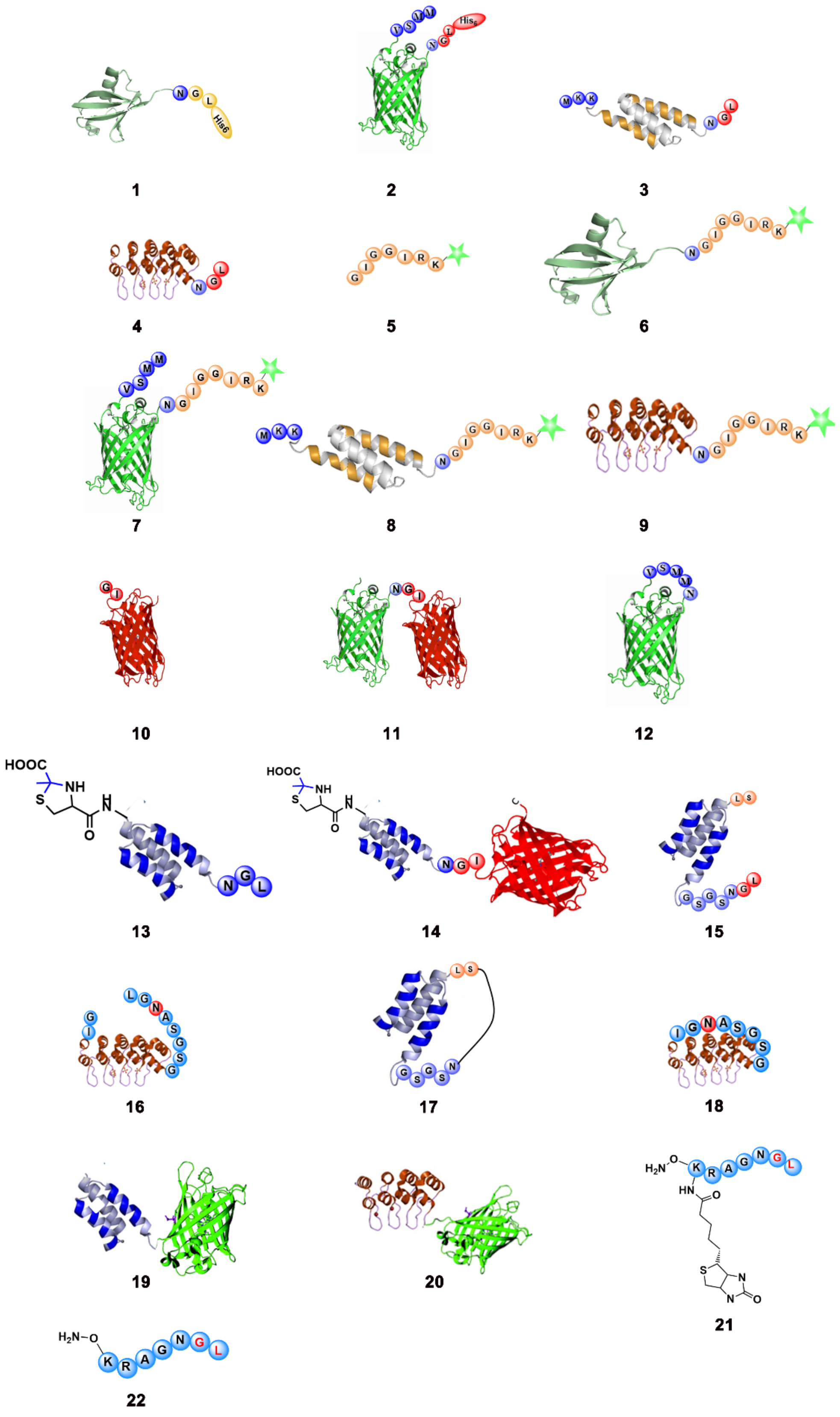

Figure S1. Numbering and illustrative structures of peptide/protein compounds used or prepared in this study. The green star is 5(6)-carboxyfluorescein coupled through its carboxyl group to the side-chain amine of a lysine residue.

b. VyPAL-mediated ubiquitin labeling

In this experiment, we used the fluorescein-peptide **5** containing a Gly-Ile dipeptide motif at the N terminus as the nucleophile substrate and Ub-NGL **1** as the acyl donor substrate for VyPAL2 (Figure 1 in main text and Figure S2A). The reaction was performed by mixing 100 µM of protein **1** with 500 µM fluorescein-peptide **5** and 100 nM VyPAL2 in phosphate buffer (pH 7.4) at 37 °C. The labeled ubiquitin was isolated by HPLC and its molecular weight was confirmed with ESI-MS (Figure S2B). The reaction gave the ligation product in approximately 80%.

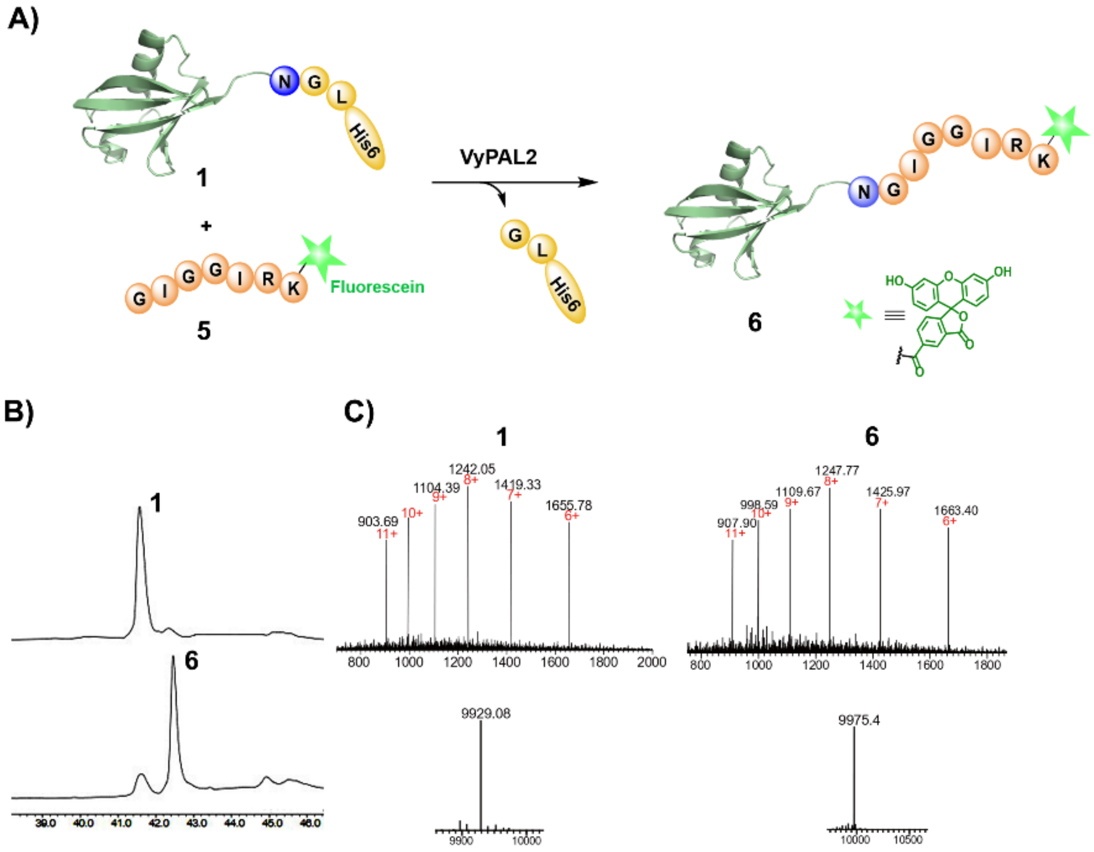

Figure S2. VyPAL-mediated ubiquitin C-terminal labeling. **A**) Scheme of VML between Ub-NGL **1** and peptide **5**. **B**) HPLC monitoring of ligation reaction and ESI-MS characterization of the fluorescent peptide **5**-labeled Ub **6**. Ub-NGL **1:** calcd mass 9928.5 and obsvd mass 9929.1; product **6:** calcd mass 9974.1 and obsvd mass 9975.4.

c. VyPAL-mediated sfGFP labeling

The sfGFP labeling experiment was performed using the fluorescein-peptide **5** and sfGFP-NGL-His$_6$ **2** (Figure S3A). The reaction was performed by adding VyPAL2 (100 nM) to the mixture of 100 µM sfGFP-NGL-His$_6$ **2** and 500 µM fluorescein-peptide **5** in phosphate buffer (pH 7.4) at 37 °C. Aliquots of the reaction mixture were taken out at different time points for SDS gel analysis. The samples were subjected to fluorescent SDS gel electrophoresis after treatment with 10 mM tris(2-carboxyethyl)phosphine (TCEP) which destroyed the fluorescence of the GFP protein. The observed fluorescence of the labeling product **7** was due to fluorescein on the peptide label (Figure S3B). HPLC

analysis showed a yield of 70% of the labeling product at 30 min (Figure S3B). The labeled sfGFP **7** was isolated by HPLC and its molecular weight was confirmed by ESI-MS (Figure S3C).

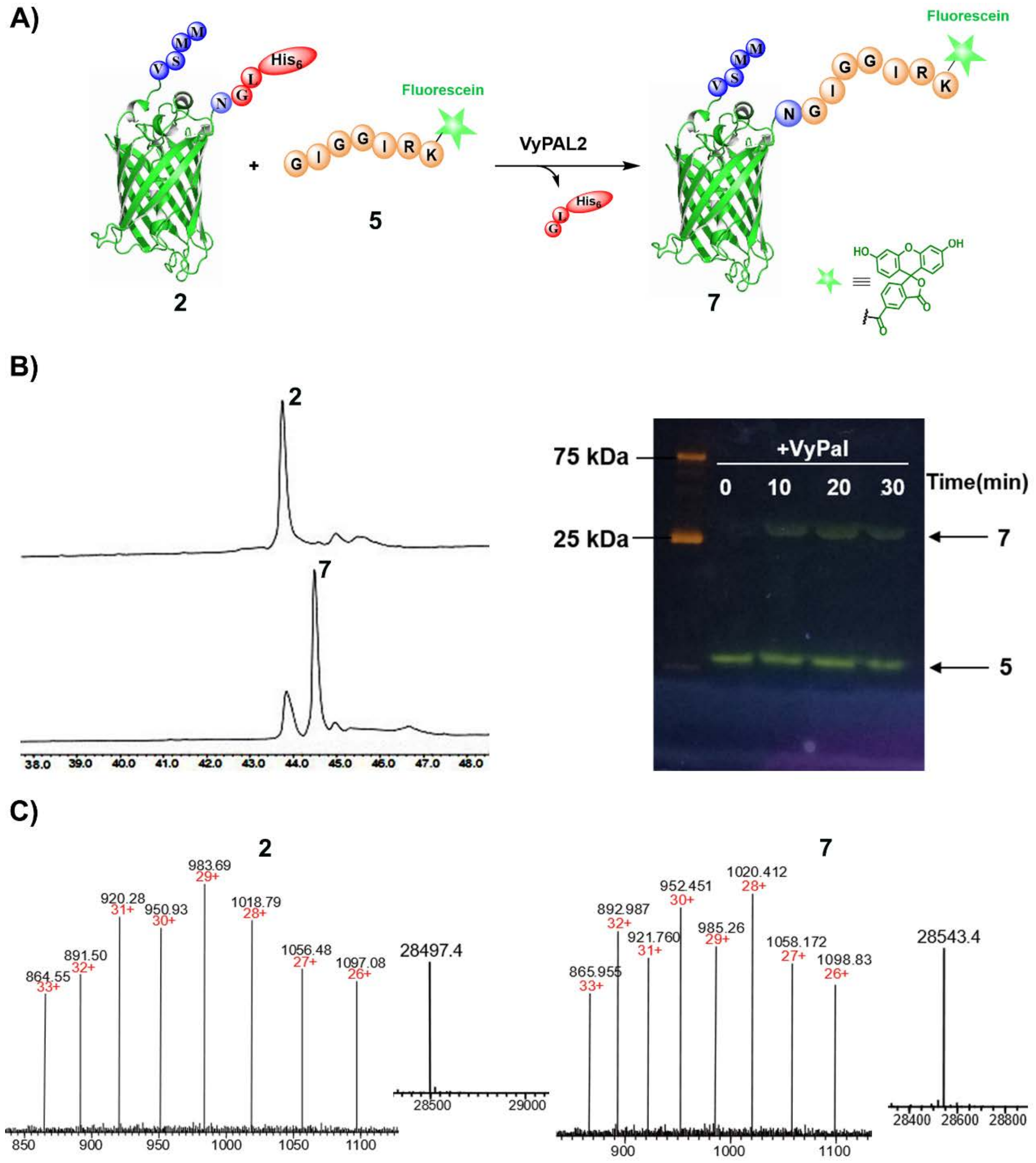

Figure S3. A) Schematic diagram of sfGFP labeling with fluorescein-peptide **5** mediated by VyPAL2. **B)** HPLC and fluorescent SDS analysis of the labeling reaction. Left: HPLC monitoring the reaction at 30 min; Right: SDS gel of the reaction mixture at different time points analyzed by fluorescence imaging. **C**) ESI-MS analysis. sfGFP-NGL **2:** calcd mass 28494.0 and obsvd mass 28497.4; product **7:** calcd mass 28540.1 and obsvd mass 28543.4.

d. VyPAL2-mediated DARPin labeling

DARPin **4** which has the C-ter NGL-His$_6$ tag was prepared by recombinant expression in E. coli cells. 50 µM of DARPin **4** was reacted with 250 µM of peptide **5** in the presence of 50 nM VyPAL2 to give product **9**. The reaction was monitored by reverse-phase HPLC using a C4 column (3.6 µ particle size, 4.6 X 150 mm). As shown in Figure S4B, the reaction was about 90% completed in 40 min.

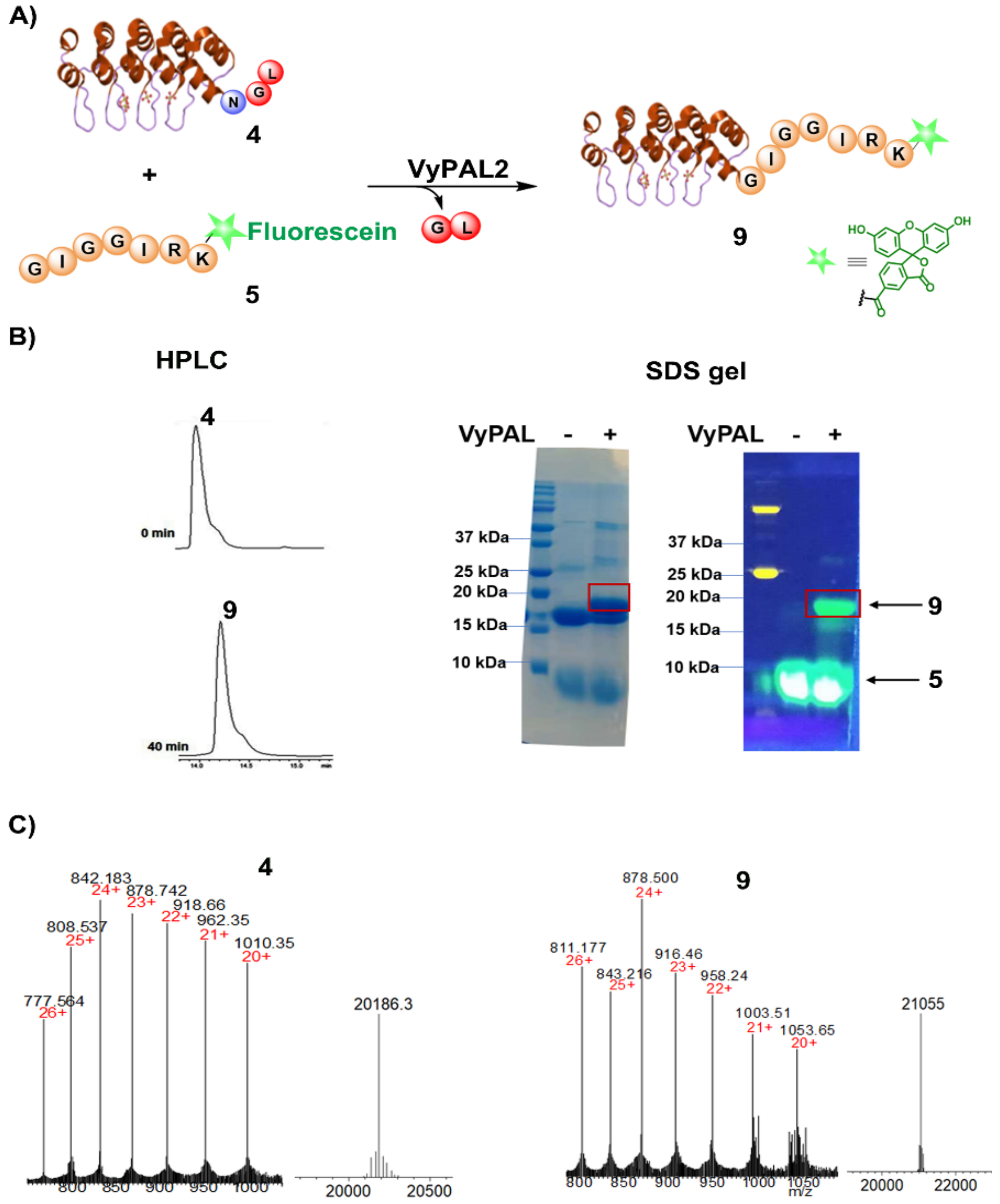

Figure S4 VyPAL2-mediated labeling of DARPin **4** with the fluorescein-pepitde **5**. **A**) The reaction scheme of VyPAL-catalyzed ligation between DARPin **4** and the fluorescein-pepitde **5**. **B**) Left: HPLC monitoring of the ligation reaction at 40 min; Right: Fluorescent SDS-page gel analysis of the labeling

reaction. **C**) ESI-MS characterization of DARPin **4** and the labelling product **9**. DARPin **4:** calcd mass 20185.7 and obsvd mass 20186.3; product **9:** calcd mass 21059.2 and obsvd mass 21055.6.

e. VyPAL2-mediated affibody and mCherry inter-protein ligation

To perform the reaction, to the mixture of Thz-Z_{EGFR}-NGL **13** (80 μM) and GI-mCherry **10** (40 μM) in phosphate buffer (pH 7.4) was added VyPAL2 (200 nM). The reaction temperature was maintained at 37 °C. The reaction was monitored at 0, 5, 10, 15, and 30 min by subjecting the reaction mixture to fluorescent PAGE gel analysis (Figure S5).

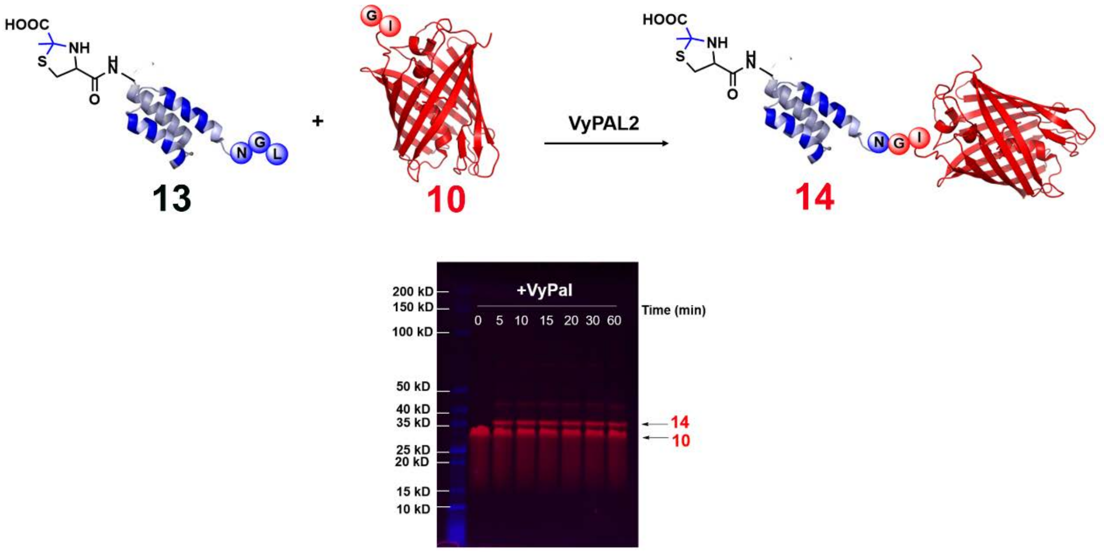

Figure S5. Fluorescent gel analysis of Z_{EGFR}-NGL **13** ligating with GI-mCherry **10** by the catalysis of VyPAL2. After the addition of VyPAL2, aliquots of reaction mixture were taken out at different time points and frozen, and at the end subjected to SDS-PAGE gel electrophoresis. The gel was analyzed by fluorescent imaging using ChemiDoc MP Gel Imaging System (Bio-Rad). For marker and mCherry visualization, 590/110 and 602/50 filters were used, respectively.

f. VyPAL2-mediated sfGFP macrocyclization

sfGFP **2** with a C-terminal NGL motif and an N-terminal MM dipeptide motif was used for cyclization (Figure S6A). 100 μM of **2** was mixed with 50 nM of VyPAL2 in phosphate buffer (pH 7.4) and incubated at 37 °C. The reaction was about 65% completed at 20 min as monitored by HPLC and PAGE-gel analysis. At 60 min, the reaction was about 90% complete as shown by PAGE-gel analysis (Figure S6B). The cyclic product was confirmed by ESI-MS (Figure S6C).

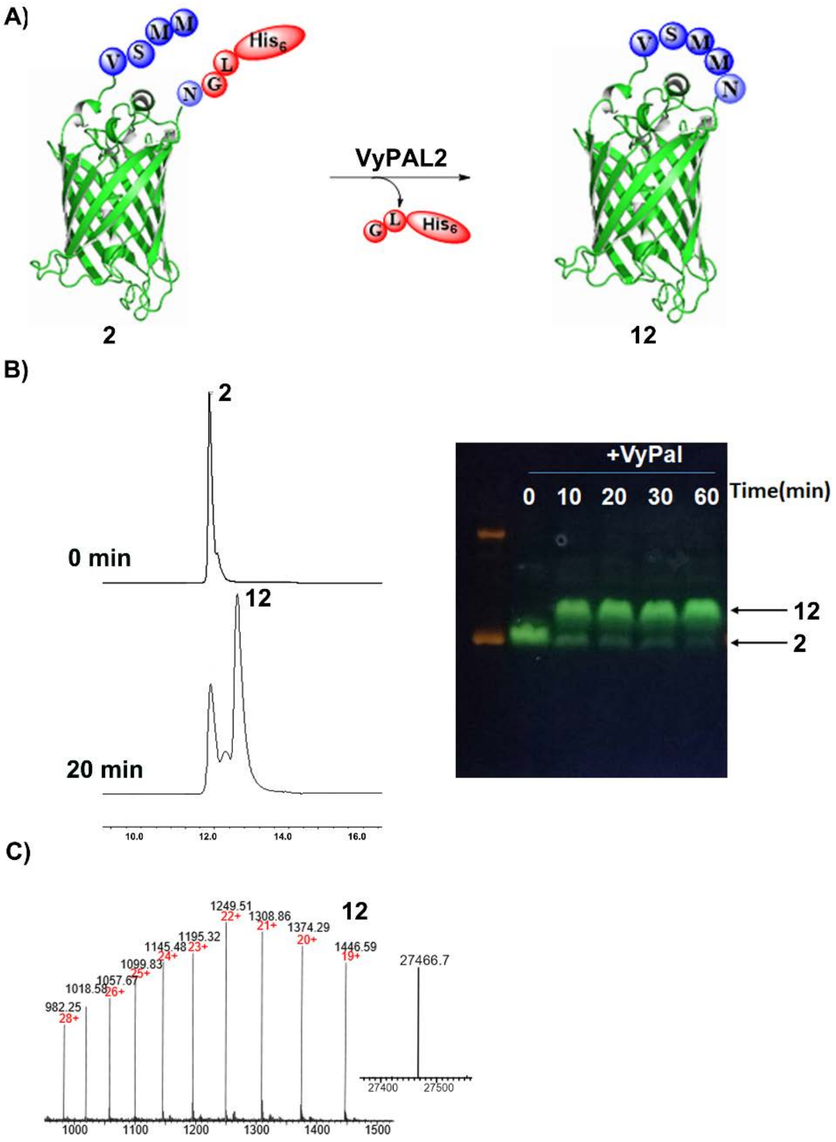

Figure S6. VyPAL2-mediated macrocyclization of sfGFP **2**. **A**) Scheme of VyPAL2-mediated sfGFP **2** cyclization. **B**) Monitoring and analysis of VyPAL-mediated sfGFP cyclization using HPLC and fluorescent SDS PAGE gel. **C**) ESI-MS analysis. cyclic product **12**: calcd mass 27466.6 and obsvd mass 27466.7.

g. VyPAL2-mediated DARPin macrocyclization

DARPin-NGL **16** was designed to contain an N-terminal dipeptide GI which is known to function as a good nucleophile. The cyclization reaction was done by adding 1 µM of VyPAL2 to 50 µM of GS-DARPin-NGL in phosphate buffer (pH 7.4) at 37°C for 90 min, which gave a yield of about 85% of the cyclized protein **18**, as shown by HPLC analysis. The cyclic protein was confirmed by ESI-MS analysis (Figure S7B).

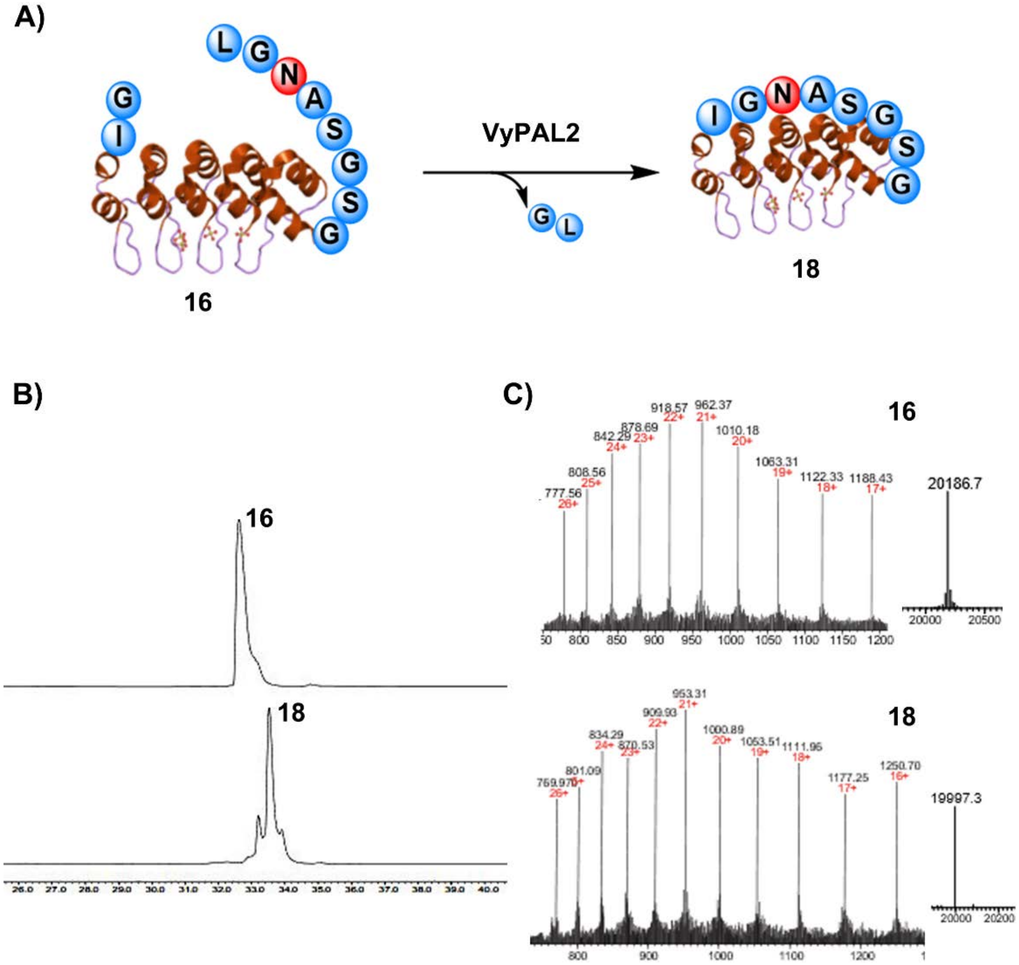

Figure S7. Cyclization of DARPin **16** catalyzed by VyPAL2. **A)** Schematic illustration of VyPAL2-mediated cyclization of DARPin **16**. **B)** HPLC and ESI-MS analysis of DARPin cyclization reaction. DARPin **16:** calcd mass 20190.1 and obsvd mass 20186.7; Product **18:** calcd mass 19996.8 and obsvd mass 19997.3.

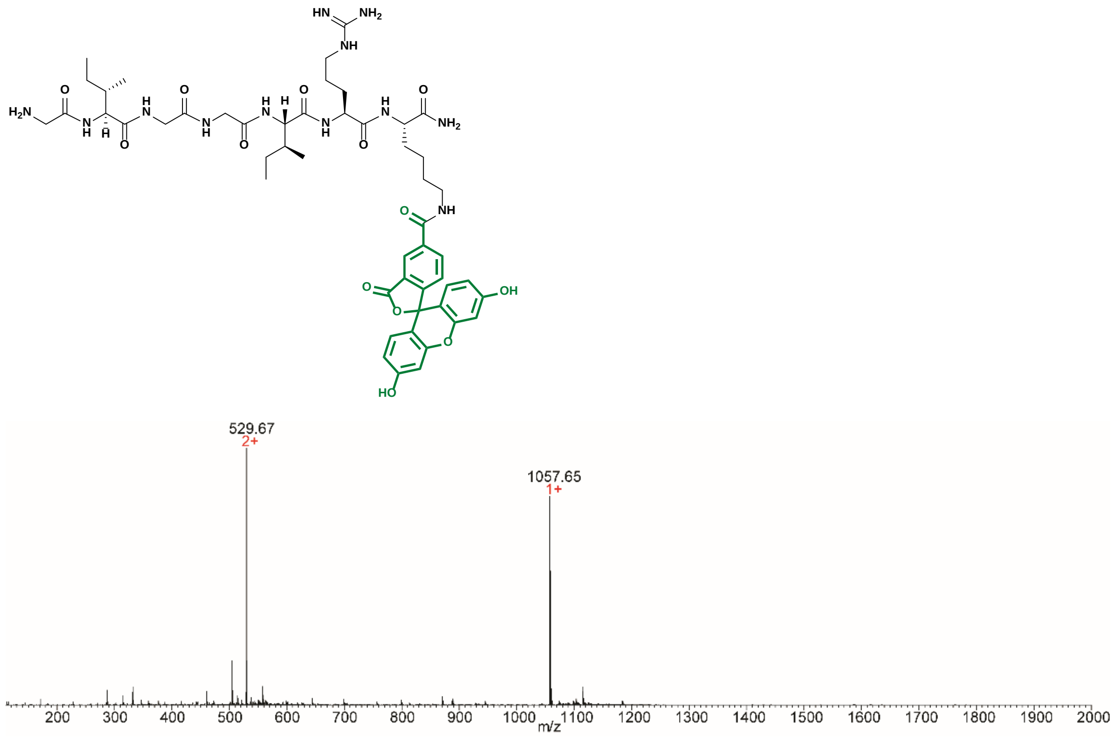

Figure S8. ESI-MS of peptide **5**. ESI-MS of pepitde **5:** ESI-MS: 1057.65 (observed), 1057.18 (calculated).

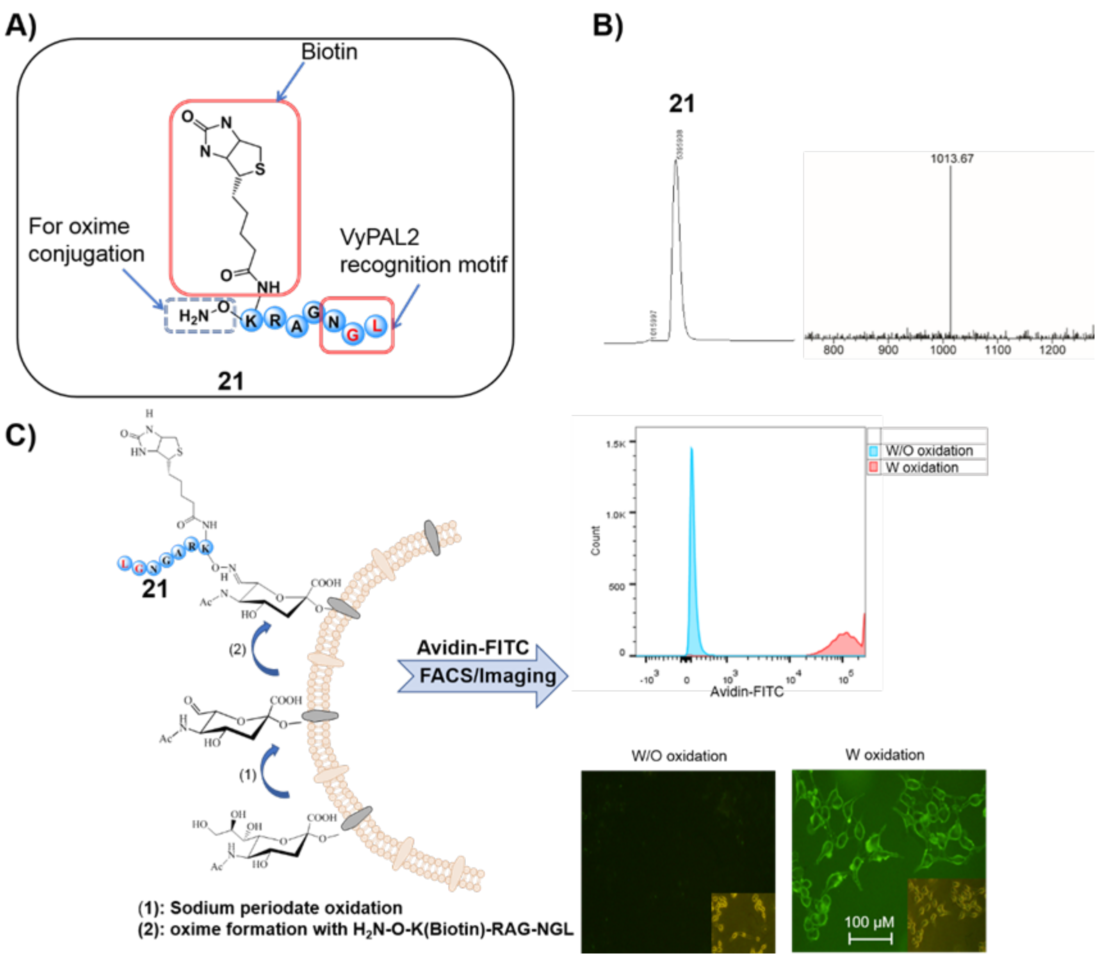

Figure S9. Characterization of peptide **21** and A431 cell surface conjugation with **21**. **A**) Structure of peptide **21**. Peptide **21** contains the –NGL tripeptide motif at the C terminus as the VyPAL2 substrate and an N-terminal aminooxyl functional group which can react with an aldehyde through imine formation. **B**) HPLC and ESI-MS characterization of peptide **19**. **C**) Evaluation of peptide **21** conjugation with pre-generated cell-surface aldehydes using flowcytometry analysis. Left panel: Schematic illustration of the procedure. (1) Sodium periodate oxidation of sialic acid residues on cell-surface glycans (by treatment with 10 mM sodium periodate in PBS at 0 °C for 5 min). (2) Imine formation (oxime conjugation) by treating the A431 cells with 1 mM of the aminooxyl-peptide **21** to anchor the peptide - a VyPAL2 substrate - onto the cell surface. Right upper panel: flowcytometry analysis of Avidin-FITC treated A431 cells decorated with **21**. Right lower panel: fluorescent microscopy analysis of cells after treatment with or without sodium periodate. Both groups of the cells were treated with avidin-FITC and washed with PBS for 3 times before subjecting to flowcytometry analysis.

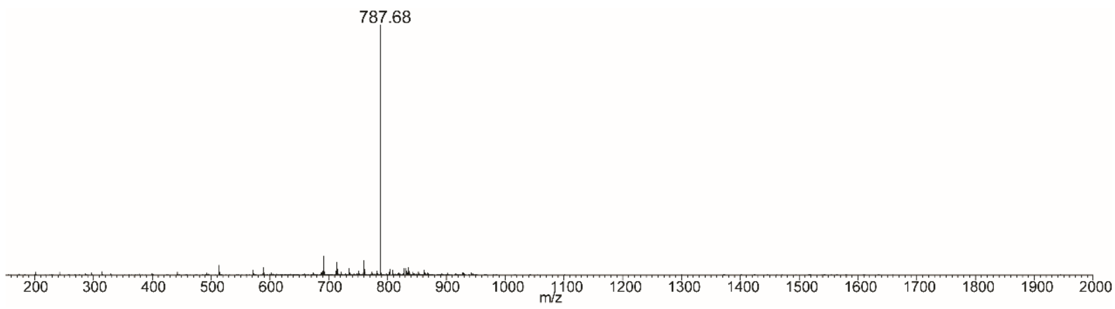

Figure S10. ESI-MS analysis of peptide **22.** ESI-MS: 787.68 (observed), 787.89 (calculated).
